# Monitoring Heritage Buildings with Open Source Hardware Sensors: A Case Study of the Mosque-Cathedral of Córdoba

**DOI:** 10.3390/s16101620

**Published:** 2016-09-29

**Authors:** Francisco Javier Mesas-Carrascosa, Daniel Verdú Santano, Jose Emilio Meroño de Larriva, Rafael Ortíz Cordero, Rafael Enrique Hidalgo Fernández, Alfonso García-Ferrer

**Affiliations:** Department of Graphic Engineering and Geomatics, University of Córdoba, Campus de Rabanales, Córdoba 14071, Spain; p12vesad@uco.es (D.V.S.); jemerono@uco.es (J.E.M.d.L.); p82orcor@uco.es (R.O.C.); ig1hifer@uco.es (R.E.H.F.); agferrer@uco.es (A.G.-F.)

**Keywords:** relative humidity and temperature monitoring, cultural heritage, open source hardware

## Abstract

A number of physical factors can adversely affect cultural heritage. Therefore, monitoring parameters involved in the deterioration process, principally temperature and relative humidity, is useful for preventive conservation. In this study, a total of 15 microclimate stations using open source hardware were developed and stationed at the Mosque-Cathedral of Córdoba, which is registered with UNESCO for its outstanding universal value, to assess the behavior of interior temperature and relative humidity in relation to exterior weather conditions, public hours and interior design. Long-term monitoring of these parameters is of interest in terms of preservation and reducing the costs of future conservation strategies. Results from monitoring are presented to demonstrate the usefulness of this system.

## 1. Introduction

Cultural heritage is our legacy from the past and, depending on its nature, defined climatic conditions need to be maintained for optimal conservation and to avoid deterioration [1]. Therefore, monitoring microclimatic conditions is important in preventing deterioration of artwork or structural architecture [2,3]. Microclimatic monitoring is a useful tool for the protection of works of art in museums or archives [4], as well as for monitoring structures hosting cultural heritage, such as frescos [5]. Its usefulness has also been demonstrated in measuring fluctuations in temperature, relative humidity, and in its capacity to alert of the presence of soluble salts or microbiological agents, among others [6].

The interest in monitoring climatic parameters using technological components in cultural heritage is growing [7]. Further methods are being employed more and more frequently to study and preserve cultural properties [8,9].

In this context, climatic variables in cultural heritage sites have been successfully monitored [10,11], to develop energy savings strategies in museums [12], analyze the impact of lighting and people [13], control physical parameters of interior environments of museums [14] or tombs [5], among others. In general, extreme variations in temperature or relative humidity may cause damage or deformation to heritage Works, like wood panels or frescoes [15]. Therefore, early detection of anomalies is essential to their preservation [16].

Recent progress in electronics, wireless communications and the production of small-sized sensors provide new opportunities to monitor and control homes, cities, crops, and the environment [17]. The number of studies demonstrating the value of the preservation and care of cultural heritage is increasing as well [18,19]. On conserving cultural heritage, the application of technology offers useful advantages [20]. It allows the registration of data with a frequency of data per day, per hour, or more [18,21]. Monitoring in higher frequencies (1 datum/min) is interesting because of the greater recording potential of valuable information which leads to greater accuracy for statistical analysis [22]. Additionally, current electronic devices are smaller and more accurate, and easier to keep hidden from spectators, permitting focus to be kept on the artwork [23]. 

Some authors use commercial data loggers like Hobo [24], DS1922L [25], or DS1923 [26]. The hardware and software of these commercial devices are not open source and, therefore, cannot be modified [27]. Open source hardware (OSH) can be an alternative when customization is necessary. Although OSH is quite new [28], it is being used for different applications in agriculture [17], pharmacology [29], and monitoring radiation [30], among others. In reference to cultural heritage, some researchers have developed data acquisition systems, for example, [31] designing a system for the remote control analysis of wall paintings using Arduino technology.

This manuscript describes a system which registers microclimate parameters to be used in preventive conservation of heritage buildings. Its practicality is demonstrated in its application to the Mosque-Cathedral of Córdoba (Spain).

The article is divided in the following sections: in Section 2, we present a brief description of the study área; in Section 3, we describe the technology, materials, and methods used to set up the monitoring system; in Section 4, some field tests and their results are presented, followed by a conclusion.

## 2. Description of the Mosque-Cathedral of Córdoba

**Figure 1:**
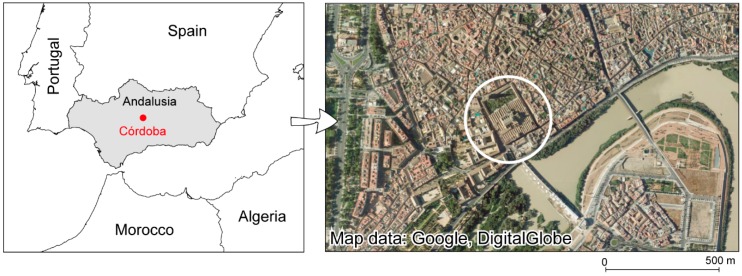
Location of the Mosque-Cathedral of Córdoba.

Córdoba (Spain) was the capital of al-Andalus for three centuries, housing the largest mosque west of the Moslem world. The Mosque-Cathedral of Córdoba (37°52′43′′ N, 4°46′46′′ W, WGS84) (Figure 1) is registered with UNESCO (United Nations Educational, Scientific and Cultural Organization) for its outstanding universal value.

**Figure 2:**
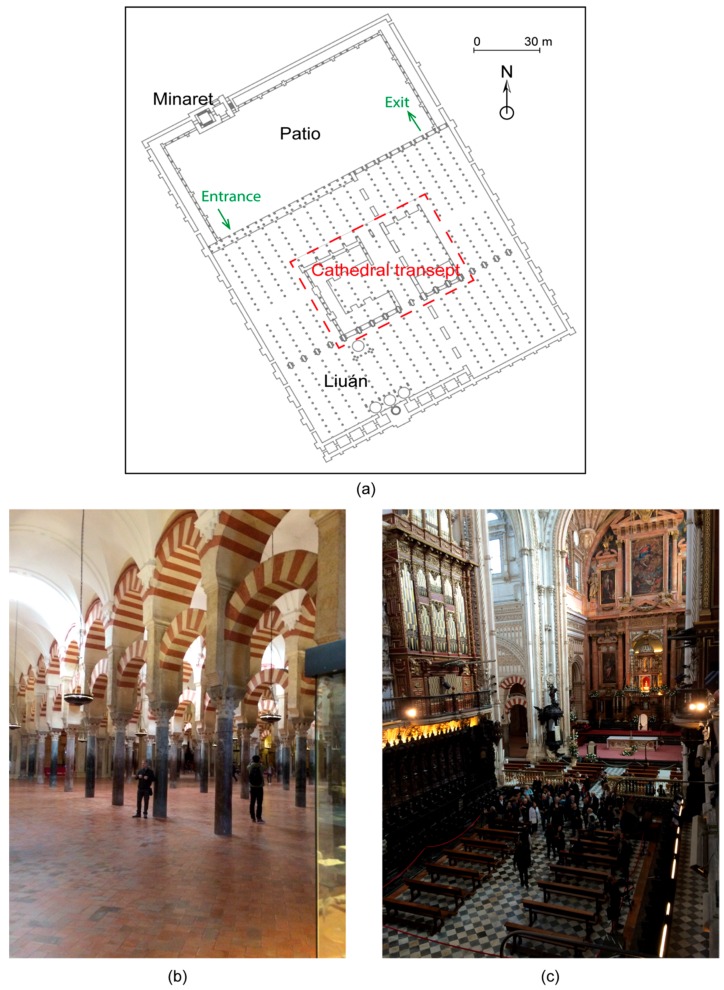
Details of Mosque-Cathedral: (**a**) key structural elements; (**b**) liuán; and (**c**) cathedral.

The Mosque-Cathedral has endured many transformations, including extensions, alterations, and reformations, both during the Moslem period and after the Christian conquest of the city. There are four principal elements in its construction (Figure 2a): the minaret, a patio (or sahn), a hypostyle hall (or liuán), and a cathedral transept. The hall, or liuán, is a rectangular space with a support system based on double-arched columns (Figure 2b). In 1521, a choir and chancel were built in the center of the Mosque-Cathedral and together form a Latin cross (Figure 2c). Visitors access the liuán through a door at the top left and exit through the top right (Figure 2a).

All microclimate stations (MCSs) developed in this study have been discreetly installed inside the liuán and transept with the exception to one exterior MCS which was placed on the roof of the the Mosque-Cathedral.

## 3. Materials and Methods

### 3.1. Monitoring System

**Figure 3:**
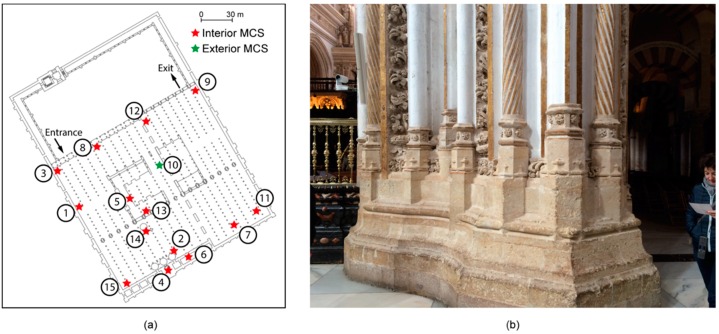
(**a**) Distribution of MCSs in the Mosque-Cathedral and (**b**) a photograph of the transept wall.

A total of 15 MCSs were installed, 14 in the interior of the Mosque-Cathedral and one on the roof to monitor outdoor weather conditions (Figure 3a). All MCSs were based on the Arduino platform (www.arduino.cc). Arduino is a single-board microcontroller based on easy-to-use hardware and software. It is a descendant of the open-source Wiring platform and is programmed using a Wiring-based language similar to C++. Arduino boards can be purchased pre-assembled or as do-it-yourself kits with ready access to hardware design information.

In this study, the Arduino UNO R3 [32], a microcontroller board based on the ATmega328 microcontroller, was used. It has the minimal necessary requirements for the study. It contains 14 digital input/output pins, six of which can be used as pulse width modulation outputs. Moreover, it has six analogue inputs that provide 10 bits of resolution. The ATmega328 also has a 16 MHz crystal oscillator, a USB connection, a power jack, an ICSP (In Circuit Serial Programming) header, and a reset button. It has 32 KB of flash memory with 0.5 KB used for boot loading, library storage, as well as the storage of the serial numbers of the sensors and the main program, 2 KB of SRAM and 1 KB of EEPROM.

In the first attempt, a wireless sensor network (WSN) was designed to automatically connect the nodes to each other via a wireless link for remote data transmission. However, the wireless link did not work properly because of the thickness of the transept wall, which had a width greater than three meters (Figure 3b). Therefore, each node was set up to work in standalone mode and data were manually downloaded.

**Figure 4:**
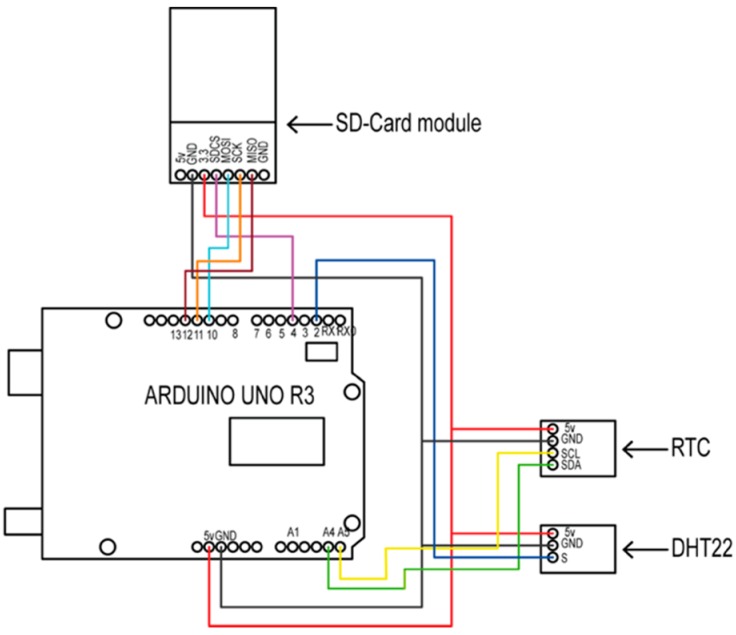
Wiring diagram of a sensor network node with sensors.

Figure 4 shows the wiring diagram of a MCS. The role of the microcontroller ATmega328 was to manage, arrange, and store the onboard sensor data. Each MCS was scalable and it was possible to include other sensors. In this study, temperature and relative humidity sensors were used. The sensors were chosen considering ISO standards 15757 and 15758 [33,34]. These standards define the procedures and instruments used for measuring temperature (T) and relative humidity (RH) in the conservation of cultural property. A summary of these standards can be found in [35], which highlights the measuring range, precision, and resolution of these sensors. According to these standards, T sensors have to have an uncertainty of 0.5 K and a resolution of 0.1 K. RH sensors have to have a typical measurement uncertainty of 5% and a resolution of 0.1%. In order to verify that sensors worked properly, they were calibrated considering environmental conditions, as in [17]. Ten measurements were taken using a five minute time interval. A PCE-222 environtmental meter (PCE Instruments Ltd., Southampto, Hampshire, UK) was used for reference. The outcome was a linear model that improved data recorded by the sensor.

The DHT22 digital sensor was used to measure RH and T [36]. It operates with a voltage supply between 3 and 5 V. It is able to measure RH between 0 and 100%, with an accuracy of ±2% and ±5%, usually at the end of a measuring range, and T with a range between 233.15 to 353.15 K, with an accuracy better than ±0.5 K. It provides a calibrated digital output signal, assuring its reliability and stability, using humidity-sensing technology and an exclusive digital signal collecting technique. Each sensor is temperature compensated using a calibration coefficient stored in the On-Time Programmable (OTP) memory memory.

Each measurement was temporally referenced with a DS1307 real-time clock [37]. It operates with a supply of 5.0 V and can operate between 233.15 K and 358.15 K. This device is a low-power, full binary-coded decimal clock/calendar with 56 bytes of non-volatile SRAM. It uses the I2C protocol to communicate with the microcontroller and an external battery to guarantee the operation if main power fails.

Each MCS operated in standalone mode. For that reason, each MCS stored the information on an 8 GB SD card and the data was manually downloaded periodically. A 220–5 V transformer in the USB port was used to power each MCS. Due to this, each MCS was installed near a 220 V electric power connection. The mechanical structure of each node used was an outdoor waterproof case whose ingress protection was equal to 68. Sensors were installed with adequate ventilation and T and RH values were not altered by the operation of the microcontroller board. 

### 3.2. Data

All MCSs were installed at the end of December 2015 with a data acquisition frequency of one data package every 2 min. Thus, each MCS recorded approximately 21,600 data packages per month. Each data package reported the time and date of acquisition, MCS identification, T, and RH.

Data were processed in two stages, first to detect anomalous and group data in time intervals equal to 5 min and, second, to generate maps and graphs of continuous spatial distribution for each parameter. 

In the first stage, an algorithm detected and eliminated anomalous data caused by an error in data collection. Maximum variation allowed per two min was set up to equal ±5 K of T and ±5% of RH, as in [38]. This anomalous behavior may be due to the sensor itself or to external agents. In the case of the sensor, in addition to the accuracy and precision of the sensor itself, it is possible that some components, under certain T or RH conditions, might not work properly. Externally, installed MCSs can be exposed to conditions (people, open doors, etc.) that could generate significant variations of T or RH for a short time which are not significant. Therefore, the percentage of eliminated data was 1.2%. Subsequently, data were filtered with a temporal frequency of 5 min allowing data to be collected continuously in case a measurement was removed. 

In the second stage, data were processed to generate continuous maps showing the spatial distribution of T and RH throughout the Mosque-Cathedral and evolution graphs of T and RH. Triangulation was generated and, afterwards, a continuous grid was interpolated with a spatial resolution equal to 0.5 m for each parameter every 5 min.

Analysis and processing of data to generate the maps and graphs were done using algorithms developed through MATLAB (Mathworks, Natick, MA, USA).

## 4. Case Study and Experimental Results

On 1 January 2016 all MCSs were installed and began recording data autonomously. From then on, data from each individual MCS were downloaded every week and processed to remove possible outliers. Afterwards, filtered data were used to generate products of interest, like distribution maps of T and RH, and evolution graphs and plots of T and RH comparing individual MCSs.

Every five minutes T and RH distribution maps were generated from MCSs installed inside the Mosque-Cathedral. These individual maps of T and RH were compiled into video frames to generate an animation of the evolution and dynamics of these parameters over time. It also highlighted the differences of T and RH, while delimiting superfluous behavior and, therefore, facilitated technical analysis.

**Figure 5:**
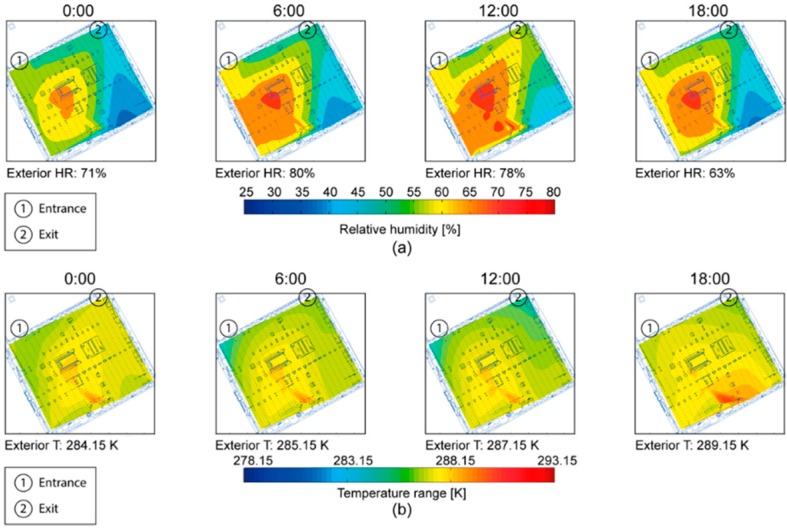
Evolution of distribution: (**a**) relative humidity; and (**b**) temperature on 10 March 2016.

The generated maps reflected the behavior of RH and T and how these parameters were influenced by the interior design of the Mosque-Cathedral, exterior weather conditions, and visiting hours. Figure 5 shows a distribution map measuring (and demostrating typical) T and RH values every 6 h on 10 March 2016. Figure 5a shows RH started high in the center of the Mosque-Cathedral and, as the day progressed, RH increased and expanded from this point to the lower left corner. Meanwhile, the right side of the building showed lower values. Figure 5b shows the evolution of T. In this case, higher temperatures were concentrated in the center and bottom half while having a radial distribution.

In general, both T and RH increased as the inner interior of the building was approached. Moreover, these maps show the transept of the cathedral directly influences the dynamics of the RH. Furthermore, the higher temperature area appears in the lower region of the building. This location is home to an exhibition area of culturally interesting items which are protected by illuminated glass cases whose spotlights may be the cause of these higher temperatures. Moreover, visitors may increase T and RH values in this area in certain moments due to the fact that they remain longer in this exhibition area than in the rest of the Mosque-Cathedral.

A piori, the behavior of T and RH inside the Mosque-Cathedral is influenced by exterior weather conditions, but the degree of influence is unknown. Due to this, a comparison of the exterior MCS and interior MCSs was done. Table 1 shows statistics of T and RH based on data registered by the MCSs installed in the corners of the interior of the Mosque-Cathedral (MCSs 3, 9, 11, and 15) and outside (MCS 10). Analyzing T, MCS 9 (upper right corner) registered values quite similar to those registered by MCS 10. MCS 9 showed a mean value equal to 285.95 K and a standard deviation equal to ±1.5 K. On the other hand, MCS 3 (upper left corner) registered a mean value equal to 286.45 K and a lower standard deviation equal to ±0.9 K. MCSs 11 and 15 registered similar mean values: 287.05 K, but MCS 11 showed the most stable behaviour with a standard deviation equal to ±0.7 K. The behavior of T inside and outside the Mosque-Cathedral were similar when considering mean values, but interior conditions of the Mosque-Cathedral were more stable, showing lower values of standard deviation compared to exterior conditions. On the other hand, although interior MCSs showed similar mean values of T, there were differences in standard deviation. In the first analysis, MCSs installed on the left side had a standard deviation lower than ±1 K, meaning that the dynamic temperature range is lower in this area of the Mosque-Cathedral. Taking RH into account, there are more evident differences between the interior and exterior. Outdoor conditions showed a broad range of RH, the mean value was equal to 79.4%, and the standard deviation was ±12.7%. Inside the Mosque-Cathedral, MCS 15 (lower left corner) registered the highest mean RH value: 63%, and the most stable behavior with a standard deviation equal to ±5.0%. The lowest values of RH occurred in the lower right corner, MCS 11, with a mean value equal to 51.0% of RH and a standard deviation of ±8.0%. Intermediately there were MCSs installed in the upper area, MCS 3 and MCS 9, with a RH close to 56%.

**Figure 6:**
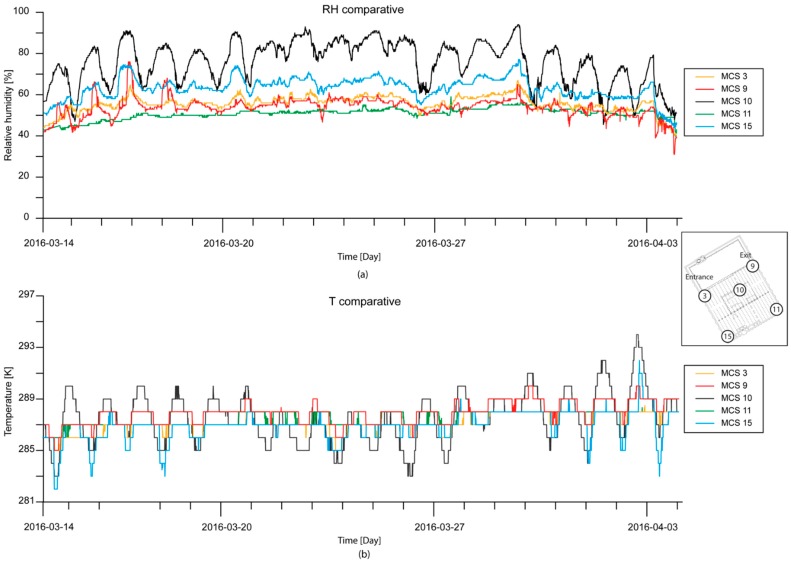
Example time series graph comparing the evolution of (**a**) RH and (**b**) T of the exterior weather conditions (MCS 10) and interior microclimate conditions of the Mosque-Cathedral (MCSs 3, 9, 11, and 15).

Figure 6 shows an RH and T time series comparison of exterior weather conditions and interior microclimatic conditions, specifically, comparing MCS 10 (exterior) against MCS 9 (interior), which is nearest to the exit, MCS 3 (interior), which is nearest to the entrance, MCS 11 (interior), which is at the lower right corner, and MCS 15 (interior), which is furthest from exterior influences (see the distribution of MCSs in Figure 3a). 

In considering RH (Figure 6a), exterior MCS 10 showed (black line), in general, cyclical night and day behavior of RH. Between 21 and 26 March 2016, the differences between night and day are not clearly reflected because it was raining during this period and, thus, RH showed stable behavior. In the case MCS 15 (blue line, Figure 6a) was defined by a line close to the minimum values of the RH of the exterior MCS 10. In this case, the evolution of RH does not define a pattern as in the above case, but instead reflected more stability. It should be noted that there were some peaks of higher values of RH in the beginning of the time period unrelated to external weather conditions registered by MCS 15 from the 16–17 March 2016. MCS 11 (green line, Figure 6a) recorded the lowest RH values while MCS 9 (red line Figure 6a) and MCS 3 (orange line, Figure 6a) recorded intermediate values between MCSs 15 and 11. However, analysis of these data should take into account, as shown in Figure 5, the different behaviors between the left and right side of the building.

Regarding T, exterior MCS 10 (black line, Figure 6b) again showed cyclical behavior. MCS 9 displayed similar results, but with reduced differences between maximum and minimum values of T (red line, Figure 6b). Moreover, maximum T registered by MCS 9 are close to those registered by the exterior MCS 10. This was not the case with the minimum values. These temperatures are linked to night periods, when the building is closed, showing that the building keeps a stable T. MCS 15 (blue line, Figure 6b) also showed similar behavior to MCS 9, but, in this case, maximum values of T were lower than those registered by MCS 9. MCS 3 (orange line, Figure 6b) and MCS 11 (green line, Figure 6b) showed intermediate behaviour between MCS 9 and MCS 15. 

As with the RH parameter, the heritage building maintains constant behavior. These graphs indicate that, in general, the innermost part of the building is cooler to its respective exterior conditions, probably due to the large stone walls that surround it.

**Figure 7:**
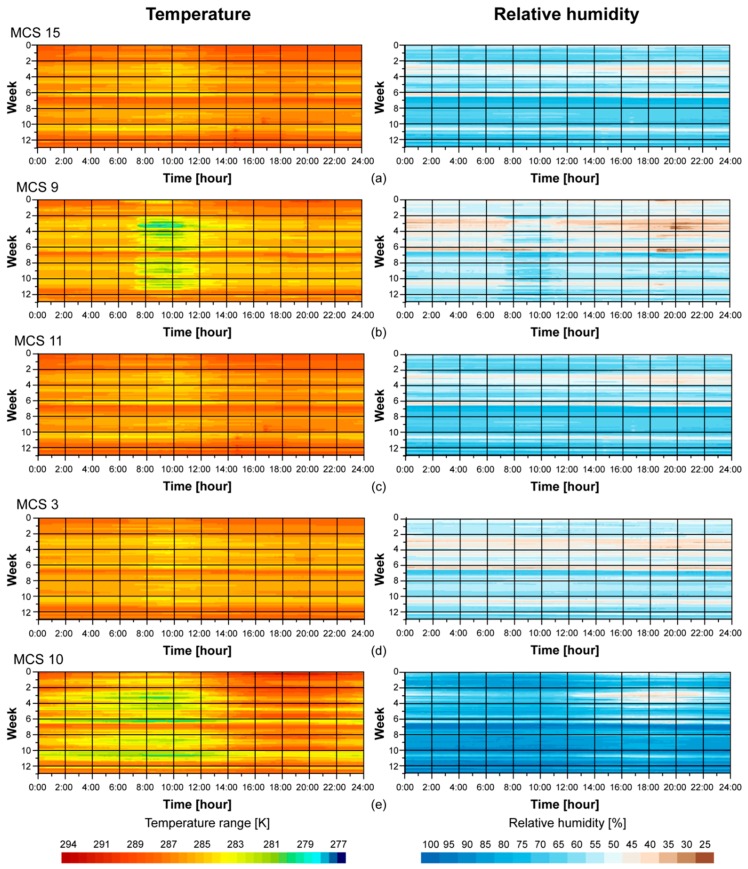
Time plot of RH and T every 5 min of interior MCSs: (**a**) MCS 15; (**b**) MCS 9; (**c**) MCS 11; (**d**) MCS 3 and (**e**) exterior MCS 10.

Along with the time series graphs of RH and T, we generated plots of the evolution of RH and T for each individual MCS. These plots show the evolution of RH and T of individual days and along a three and a half month period, from 1 January to 15 April 2016 (Figure 7). In these plots, the X-axis represents time, 0:00 to 24:00, and the Y-axis represents the day of the year. These plots were used to make a global visual comparison of all the MCSs. Moreover, they were used to study individual behaviors of the MCSs. Figure 7 presents results from interior MCSs 3, 9, 11, and 15 of the Mosque-Cathedral and MCS 10 (exterior). 

In general, MCS 15 (Figure 7a) registered higher temperatures and had a more stable range of temperatures than the other MCSs. The maximum hourly average was 287.75 K at 17:00. This MCS registered higher T values than MCS 10 with a maximun mean difference of 275.35 K at 08:00. From this moment, differences started to decrease progressively until 17:00, at which time exterior and interior T values were equal until 20:00, then MCS 15 started to register higher T values. MCS 11 (Figure 7c) showed a similar behaviour as MCS 15, registering a maximum mean T value equal to 287.25 K at 15:00. Compared to the exterior T, MCS 11 registered a maximum difference at 10:00 equal to 274.65 K and then started decreasing to 273.15 K at 15:00. At this time MCS 11 registered cooler T values than MCS 10, with a maximum difference equal to 272.45 K at 21:00. At this time, interior and exterior T differences decreased, being equal to 273.15 K at 01:00. MCS 3 (Figure 7d), like MCS 15, showed stable behavior, with a mean value of 286.75 K all day. Finally MCS 9 (Figure 7b) showed a broad range of T values and a defined pattern. From 08:00 to 12:00, nearly every day, T values decreased from 285.45 K to 284.05 K. This pattern coincides with opening hours, when the exit door was left open. This MCS registered the most similar values to the exterior T, the maximum differences being equal to 273.15 K at 06:00 and 272.15 K at 18:00.

Analyzing RH, MCS 15 (Figure 7a) showed a reduced range in value, around 63%, and lower than those registered by MCS 10 (Figure 7e). Differences with exterior conditions covered a range of values from 20.7% at 08:00 to 10.6% at 19:00. On the other hand, MCS 9 (Figure 7b) registered values of RH from 53.1% at 20:00 to 60% at 9:00, showing unstabled behavior compared to MCS 15. The range of RH differences between MCS 15 and 9 was from 4% at 08:00 to 8.5% at 19:00. Conversely, MCS 3 showed stable behaviour like MCS 15 but with lower RH values, about 56.7%. Finally, MCS 11 registered minimum mean values of hourly RH, being equal to 46.7% at 08:00. Maximum mean hourly values were registered at 17:00, being equal to 51.1%. Both MCS 9 and 11 showed broader ranges of RH values compared to MCSs installed in the left side of the Mosque-Cathedral (MCSs 3 and 15).

During the third week of the year, interior MCS plots showed lower RH values that correlated with those reported by the exterior MCS. This behavior was more accentuated in MSC 9 (Figure 7b). In general, MCS 15 (Figure 7a) registered higher temperatures than MSC 9 (Figure 7b), and had a more stable range of temperatures. On the contrary, MSC 9 had a broad range of T values and a defined pattern. From 08:00 to 12:00, nearly every day, T values decreased. This pattern coincides with opening hours when the doors are left open. A similar pattern is observed analyzing RH plots. MSC 15 (Figure 7a) shows a reduced range value. During the third week of the year, the plot shows low RH values that correlated with those reported by the exterior MCS, again being more accentuated in MSC 9 (Figure 7b). Therefore, the resulting data from MCS 9 demonstrates a correlation between climatic conditions of the exterior and interior of the building. This effect is reduced and RH and T values are more stable as the inner interior of the building is approached.

**Figure 8:**
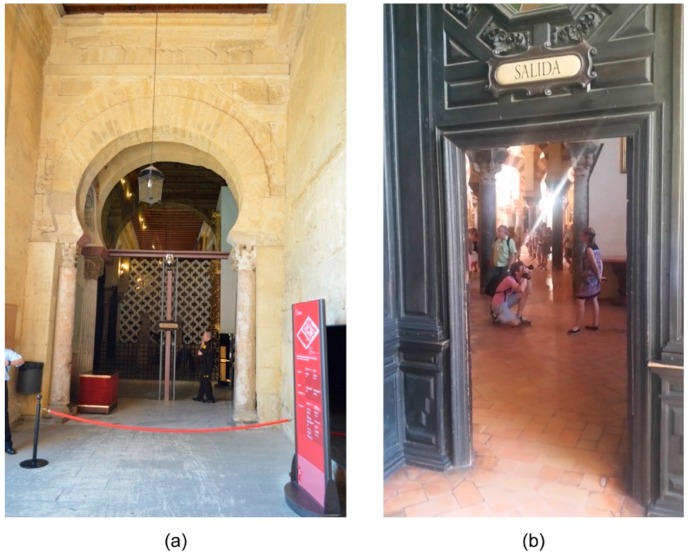
Photographs of (**a**) the entrance to the Mosque-Cathedral and (**b**) the exit.

After analyzing the results, the behavior of RH and T inside the Mosque-Cathedral are directly affected by exterior weather conditions. However, the right side of the building showed different behavior with respect to the left side. This may be due to the design of the Mosque-Cathedral’s entrance and exit. The entrance is an automatic sliding glass door which controls visitors’ access (Figure 8a) and keeps interior conditions from being affected by external weather conditions. The exit area, on the other hand, is a door which is left open during visiting hours (Figure 8b) allowing external weather conditions to be more markedly influential in this part of the building.

Thus, external air currents and the RH and T from exterior weather conditions directly affect the interior microclimate, which appears to be channeled by the right wall of the transept of the cathedral. In the case it is necessary to maintain more homogeneous microclimatic conditions inside the Mosque-Cathedral, installing an automatic sliding glass door like that at the entrance may be a suitable solution. Other interventions can be moving the visitors’ exit or delaying the opening hours. Related to the hot spot of the exhibition area, it might be appropriate to revise the lighting system in order to decrease the temperature in that area. Therefore, controlling indoor conditions can be based on a passive microclimate control as a first phase. In this case, indoor conditions would be controlled by the building itself and the outdoor climate. In practice, control should be enhanced by keeping doors and windows opened/closed. An automatic sliding glass door would maintain a more stable interior T in the upper right corner of the Mosque-Cathedral and, therefore, values of T would increase. This would increase T in the entire right side. Moreover, increasing T would increases RH values, which would be important to consider at opening hours.

Technologically, humidity could be controlled by releasing water vapor to the air if the RH is too low (humidification) or by removing water vapor from the air if the RH is too high (dehumidification). In the case of humidication, it is a method for increasing RH. It could be performed by injecting steam into the air or by the evapotation of water. In this case, T decreases because the system cools the air and, therefore, a system to heat the air may be neccesary to maintain T. Dehumifidication is a method for decreasing RH. It could be performed by condensation or adsorption. Another alternative is to develop a system of heat conservation in order to keep the relative humidity below given limits, although it may cause uncomfortable temperatures and high energy consumption. In this system, T is continuosly adjusted and not maintained at a constant value.

Zítek et al. [39] proposed a system focused on equal-sorption humidity controlling humidifier and dehumidifier devices. The system adjusts the relative indoor air humidity to a level continuosly adapting to the indoor temperature. The objective is to prevent moisture-sensitive materials from swelling or shrinking caused by changes in the absorbed moisture content. The stabilization of moisture content in order to determine the RH value is based on T and the logarithmic Herderson model, which describes the equilibrium moisture content. The proposed system is more sensitive to changes in air humidity that to variations in temperature.

Regarding the number and distribution of MCSs, this case study has separated zones of the Mosque-Cathedral, taking into account T and RH values. Future improvements of the network should be taken into account to increase the number of MCSs to allow a wireless network between nodes. Therefore, although it would be necessary to check connectivity, new MCSs should be installed in the corners of the transept walls to facilitate remote data transmission allowing the design of a real-time monitoring system to provide data for a microclimate system. Additionally, new MCSs should be installed on right side of the Mosque-Cathedral because this area showed different behaviour compared to left side and, currently, is monitored with fewer MCSs than the left side.

## 5. Conclusions

A system to monitor microclimate variables in heritage buildings using OSH was developed. OSH characteristics, such as low acquisition costs and easy customization, can eliminate obstacles associated with proprietary systems. The microclimate information described in the present work shows the behavior of T and RH inside the Mosque-Cathedral of Córdoba and its relationship to exterior weather conditions. The MCSs used have been able to quantify the effects of exterior conditions on interior T and RH of the Mosque-Cathedral, providing useful information for its conservation.

The results of these techniques have revealed how RH and T parameters had a defined behavior pattern. Those areas closest to doors present less T and RH and, as the inner interior is approached, these parameters increase. Moreover, these differences are greater when the heritage building is open to the visitors. Once it is closed, they are reduced. It was also noted that exterior weather conditions directly influence interior conditions and are more pronounced in areas closest to the exit.

The entire system has been developed using OSH. In the future, new sensors can be installed to measure other variables, such as percentages of carbon monoxide or sulfur dioxide in the air, among others, and their relationship with the volume of visitors.

## Figures and Tables

**Table 1:** Statistical summary of temperature and relative humidity statistics inside (MCSs 3, 9, 11, 15) and outside (MCS 10) the Mosque-Cathedral.

	Temperature (K)				Relative Humidity (%)			
MCS	Min	Max	Mean	SD ^1^	Min	Max	Mean	SD ^1^
3	284.15	289.15	286.45	±0.9	34.0	81.5	56.0	±7.3
9	278.15	292.15	285.95	±1.5	27.0	76.6	55.8	±8.0
11	285.15	292.15	287.05	±1.1	29.0	64.3	51.0	±8.0
15	283.15	289.45	287.05	±0.7	35.3	84	63.0	±5.0
10	279.15	294.15	286.05	±2.1	36	99	79.4	±12.7

^1^ Standard deviation.

## Abbreviations

MCS	Microclimate station
OSH	Open source hardware
RH	Relative humidity
T	Temperature
